# Magnetic Resonance Imaging Diagnosis of Choroidal Melanoma

**DOI:** 10.7759/cureus.16628

**Published:** 2021-07-26

**Authors:** Ahmad Jiblawi, Hani Chanbour, Azzam Tayba, Haissam Khayat, Khaled Jiblawi

**Affiliations:** 1 Radiology, Lebanese American University Medical Center, Beirut, LBN; 2 Medicine, Lebanese University, Beirut, LBN; 3 Radiology, Beirut Arab University, Beirut, LBN

**Keywords:** choroidal, melanoma, retinal detachment, fovea, flair

## Abstract

Choroidal melanoma has a very rare incidence in Africa and Asia, especially in the Middle East. We report the first case of choroidal melanoma in Lebanon in a 33-year-old man presenting with progressive loss of vision in his right eye. MRI showed retinal detachment with evidence of a nodular-like lesion most consistent with choroidal melanoma. The patient underwent enucleation of the right eye globe and the diagnosis was confirmed on pathology.

## Introduction

The choroid is the layer of the eyeball sandwiched between the sclera and the retina. It belongs to the uveal tract which consists of the anterior and posterior ciliary body, in addition to the iris [1]. Choroidal melanoma is the most common primary intraocular malignant neoplasm [2] and the second most common site in a list of 10 malignant melanoma sites in the human body [1]. To note, ocular melanomas, including uveal and conjunctival, make 5% of all melanomas diagnoses, 85% of which appear in the choroid. However, choroidal melanomas often go unnoticed until they become symptomatic [3]. Epidemiologically, it has an incidence rate of six cases per one million Americans per year, and an incidence rate estimated between 0.2 and 0.4 per one million in Asia and Africa, making it a rare entity that deserves to be reported [4]. We herein report the first case of choroidal melanoma in Lebanon. We emphasize on the radiological characteristics, and we perform a brief literature review concerning this rare entity.

## Case presentation

We report a case of a 33-year-old man, previously healthy, who presented for the progressive loss of vision in his right eye and events of flashes for three months. On physical exam, there was a significant decrease in vision in the right eye compared to the left eye. The visual field was normal on the left side; a significant vision loss was noted on the right side. Extraocular eye movements were normal in both eyes; no nerve palsies and no gross defects.

An MRI of the orbit and brain was performed on a 3T Ingenia Philips unit (Eindhoven, The Netherlands) in the following planes and sequences: multiplanar thin 3D FLAIR, T1 weighted images with and without gadolinium, coronal T2 weighted images, axial diffusion weighted and ADC map, axial T2 star, and additional multiplanar 3D T2 drive sequences centered to the orbits.

**Figure 1:**
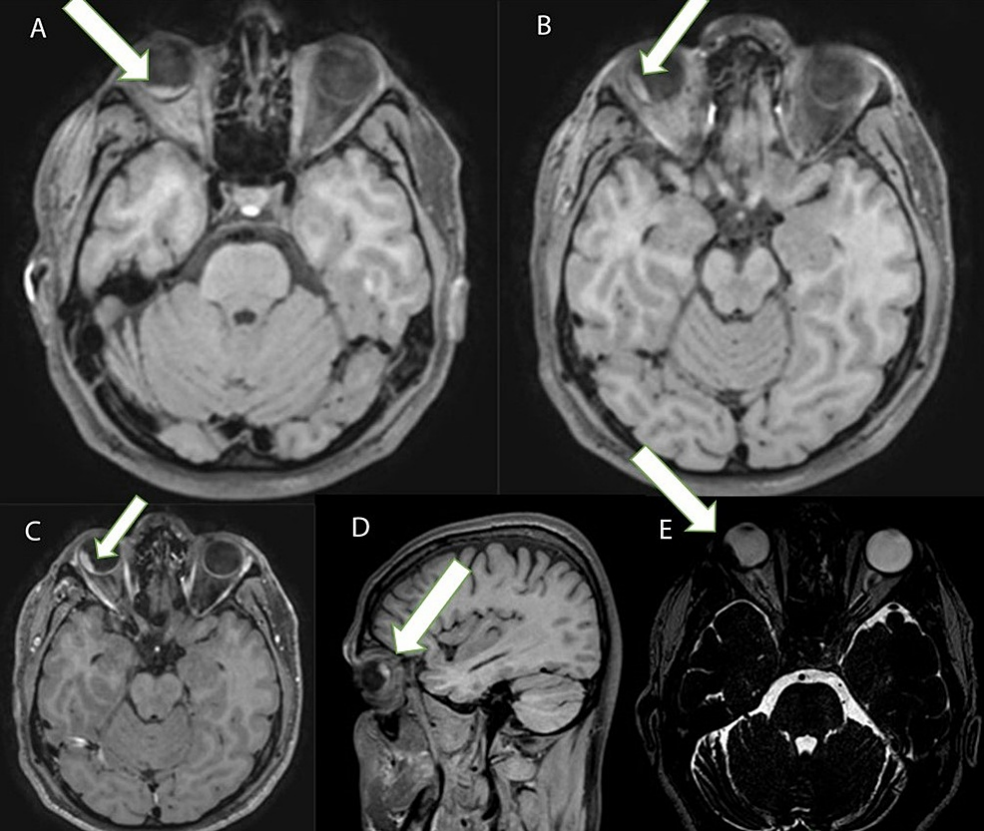
Head MRI showing T1 and T2 weighted images. A: T1 weighted image post gadolinium enhancement, axial plane: evidence of retinal detachment in the right eye globe centrally in the lower quadrants. B: T1 weighted image post gadolinium enhancement, axial plane: showing the extension of the retinal detachment posteriorly temporal in the upper quadrant with evidence of a nodular-like hyperintense lesion. Enhancement is extended more than the dimensions of the high signal lesion on T1 in the non-enhancing images. C: T1 weighted image without enhancement, axial plane: showing a hyperintense nodular-like lesion measuring 13 mm in its longitudinal axis and 5 mm in thickness. D: T1 weighted image post gadolinium enhancement, sagittal plane: showing the hyperintense nodular lesion in the temporal upper quadrant. E: T2 weighted image, axial plane: showing a nodular-like lesion of low signal. The arrow indicates melanoma location.

Results showed evidence of retinal detachment in the right eye globe basically below the fovea in the lower quadrants centrally (Figure 1A) and extended posteriorly temporal in upper quadrant (Figure 2B) where there is evidence of nodular-like lesion (estimated about 13 mm in its longitudinal axis and 5 mm in thickness) being of high signal on T1 weighted images (Figure 1A-C) and of significantly low signal on T2 weighted images (Figure 1E) and showing on top diffusion restriction. No extended enhancement is more than the dimensions of the high signal lesion on T1 in the non-enhancing images (Figure 1C). The radiology report suggested that the overall picture in keeping with an underlying choroidal pathology largely suggests a choroidal melanoma. The rest of the right eye globe, vitreous, anterior, and posterior chambers, as well as the right lens, are all of unremarkable outlines and signals. The left eye and brain were unremarkable.

The patient underwent enucleation of the right eyeball. Grossly, sections showing a black mass in the posterior choroid measured 13 mm in diameter and 8 mm in thickness. The diagnosis was confirmed on pathology which showed spindle A type malignant melanoma involving the choroid.

## Discussion

Choroidal melanomas are relatively rare and they often go unrecognized until some symptoms appear. Although non-specific, choroidal melanomas may present with retinal detachment or vitreous hemorrhage. These two symptoms carry underneath them a broad list of differential diagnoses which include choroidal hemangioma, subretinal hemorrhage, metastasis, nevus, and other benign lesions [3]. Annually in the United States, there are about six cases per million diagnosed. This incidence, which remains fairly stable over the years, consists of about one-tenth of cutaneous melanoma [5]. However, this incidence is not identical everywhere. Apparently, racial variation for the incidence of choroidal melanoma occurs, despite the fact that there are limited population-based statistics on the occurrence of choroidal melanoma according to race and ethnicity [6]. For example, the incidence in Asia and Africa was around 0.2-0.4 cases per million diagnosed yearly [4]. Choroidal melanoma in addition to other uveal melanomas affects mostly Caucasians of Northern European descent. Its incidence is extremely rare in the black population [1]. The lack of a well-developed registry for uveal melanomas prohibits an accurate estimation of the incidence of this condition in the Middle East, and as far as we know, this is the first case of choroidal melanoma to be diagnosed on MRI, reported in Lebanon [6]. Our patient belongs to an age group much lower than the mean age at which the melanoma is usually diagnosed -- he is a 33-year-old man and the mean is the mid-50s according to the Collaborative Ocular Melanoma Study (COMS) [1, 5]. Moreover, this condition has a slight male predominance [2], except in the age group from 20 to 39 years, where it is slightly predominant in women [1]. The most common presenting symptom is a progressive loss of vision, while the most common location for the tumor to appear was in the inferotemporal quadrant of the eye [4].

Choroidal melanoma is thought to develop from previously existing melanocytic nevi or from melanocytes within the choroid. The lesion begins as dome-shaped, then it slowly grows to cause secondary accumulation of lipofuscin pigments and drusen in the overlying retinal pigment epithelium. This might lead to flashes. Afterward, a break in the Bruch’s membrane would occur because of the continuous growth of the tumor, creating a collar stud or mushroom shape. Later on, the tumor might spread anteriorly, in addition to invading the retina reaching the vitreous and the extraocular space [2, 4].

On pathology, there are four distinguished categories of uveal melanomas according to the modified Callender’s classification: spindle cell melanoma, pure epithelioid cell melanoma, mixed cell melanoma, and necrotic melanoma. The first type is the most common (45%) holding the best prognosis and it is the least likely to metastasize with a five-year survival of 90%-95%. However, the epithelioid confers the worst prognosis and is more commonly observed in metastases. The mixed melanoma subtype is thought to represent a transitional phenotypic entity [2-3]. Predictors of tumor behavior associated with poor prognosis are well established; size >15 mm in the maximal linear dimension of tumor growth, necrosis, depth of invasion, scleral and ciliary body involvement, anterior and juxtapapillary locations, extrascleral tumor extension, cytologic and cytogenetic features (gain of chromosome 8 and loss of chromosome 3) [1-4]. Recently, the different morphologies are shown to have different gene expression profiles; the epithelioid variant demonstrates expression of cytokeratin, beta-catenin, epithelial cell adhesion, and basement membrane markers [3]. Our patient had the spindle A type of malignant choroidal melanoma.

Although <1% of patients have metastatic disease at the time of the initial diagnosis, COMS showed, in a longitudinal follow-up study of 2320 patients, a 10-year cumulative metastatic rate of 34%. The five-year tumor-related mortality rate was 28% in patients who had large size melanoma (>15 mm) and underwent enucleation only [5]. Also, on average, three years are required after the diagnosis of the primary tumor in order to identify a metastatic disease, however, the time to metastasis might be prolonged up to 42 years in some cases [7]. Thus, surveillance is warranted given this long-time frame in which metastasis can occur [7]. The presence in the posterior uveal location and in close proximity to the vasculature increases the tendency of such tumors to metastasize [3]. Common site of metastases includes liver (90%), lung (24%), and bone (16%) [1].

Diagnosis of choroidal melanoma is usually clinical, with emphasis on dilated fundoscopy. However, currently, imaging plays an important role in diagnosis, especially for modalities like ultrasound, CT scan, or MRI. On ultrasound, the tumor appears as a spherical mass arising or deeply embedded in the choroid, while being moderately reflective with some acoustic shadowing. In addition, a tumor as small as 3 mm might be better evaluated on ultrasound than either by CT or MRI [2]. On MRI, melanotic melanomas show high signal intensity on T1 weighted images and mildly low signal intensity on T2 weighted images. Mixed pigmentation would be suggested by heterogeneous signal intensities and amelanotic melanomas would appear slightly hyperintense or isointense on T1 weighted images. In our case, the patient was referred for imaging without having an ophthalmology consult. The patient was diagnosed by imaging and went for enucleation surgery, after which the pathology report came confirming the diagnosis. Enucleation is the standard of care for malignant choroidal melanoma. Secondary enucleation might be indicated in cases of glaucoma and tumor recurrence [4].

## Conclusions

Choroidal melanoma is a rare entity. As far as we know, this is the first case of choroidal melanoma to be diagnosed on MRI being reported in Lebanon. This case report emphasizes that choroidal melanoma is an entity that necessitates early recognition to ensure an early management.
